# West Ham Hospital

**Published:** 1902-03-15

**Authors:** 


### WEST HAM HOSPITAL.

### INTEREST OF THE WORKING MEN.

The prospect of penetrating down West Ham Lane on a cold and foggy night could hardly be an alluring one even to a dweller in Stratford, yet the governors of the West Ham Hospital, many of them working men, mustered to the number of about forty on Wednesday evening, when the annual meeting of their hospital was held. Moreover, a hearty spirit pervaded the meeting, which it was pleasant to feel, and though the room was ill-lighted and bare-looking, the keen interest of the governors, and their appreciation of the speeches more than compensated for these slight drawbacks.

The committee were a quarter of an hour late in making their appearance, and at 8.15 Mr. Thos. A. Cook (Chairman of the Committee of Management) took the chair, and was supported by Mr. Howard Tanner, J.P., Mr. G. Bush, Mr. Scrivener (secretary), and other officials. Three of the medical officers were also there.

The Chairman had no set speech to make. He apologised for not having been able to go very carefully into the report, which was only submitted late on the previous night. This lateness was owing to the annual dinner, which should have taken place last year, but only came off a week ago. The dinner, however, was phenomenally successful, and after the sum of £801 had been announced, Mr. J. R. Roberts, J.P. (the very name evoked applause from the audience) gave a princely donation of £1,200. After some comparisons between subscriptions and donations of 1900 and 1901, in which it was apparent that a good level had been maintained, the speaker said that they must not relax their efforts; he would like to see last year's amount (subscriptions £930, donations £872) doubled. The annual dinner must be kept up to the £2,000 level. He went on to speak of the rules, which were made when there was no hospital but only a dispensary; and they had since been altered. The committee had gone into them very carefully, meeting sometimes more than once a week, and they had made some further alterations. As an instance of the obsoleteness of the old rules, Mr. Cook mentioned one defining the duty of a porter:—he was to shake the mats all over the hospital. Among other improvements they had formed a special house committee, whose duty it would be to visit more often than was possible for the whole committee, and to whom the officers might refer in matters of difficulty.

Mr. Tanner having seconded, the report was adopted, and then vacancies on the committee were filled up. Two nominations had been sent to the secretary, and others were made by Governors from the body of the hall. A Mr. Marriott, though eulogised most enthusiastically for past services on the committee, from which he appeared to have retired not quite pleased with certain doings thereon, firmly refused to come forward again. "Won't you reconsider your decision?" the Chairman gently asked. "No, sir." "Can you nominate anyone else?" "I have no one." No thanks for the honour done him; simply a flat refusal. This colloquy seemed to surprise and pain those who had proposed this gentleman, but no further explanation was vouchsafed by him of this rather curious conduct, and the business went on.

Mr. Claude Worth, Ophthalmic Surgeon to the hospital, had a proposal to make, which one or two speakers complained was "sprung" upon the meeting, but Mr. Worth quite clearly explained his position, which was this: he wanted the name of the hospital changed, or at least added to, but he did not want the meeting to commit itself to any decision at the moment, and after some discussion the question was referred back to the committee. Mr. Worth's idea was that the name "West Ham" was rather misleading, since the district served was very large, and as the West Ham people practically only slept in West Ham, their work being in London, he thought London ought to support the hospital as the "East London Hospital," or the "West Ham and District East London Hospital." At any rate, he wanted East London got in somehow or somewhere. One speaker thought the combination of West Ham and East London not very euphonious, and another doubted whether there would not be great legal difficulties in the way, and Mr. Stocker, one of the medical officers, wondered whether it would make any difference to the subscriptions. The Chairman, who thought the meeting wanted a vote on the matter, tried to put one, but the meeting declined to commit itself to an opinion, and preferred leaving it to the committee for consideration.

Mr. Nicoll brought the house down by a simple suggestion that the Congregational collections might be larger if the offertory were made before the sermon rather than after; and the usual votes of thanks ended the meeting.

Nine hundred and sixty-one in-patients were treated during the year, and 82,358 attendances were made in the out-patients' department. The hon. secretary of King Edward's Fund, in sending a subscription of £300 and a donation of £600, wrote: "I am further desired to say that your hospital is reported on as being efficiently and economically worked, and in a very poor neighbourhood."

